# A Novel Objective Method for Steel Degradation Rate Evaluation

**DOI:** 10.3390/ma17246074

**Published:** 2024-12-12

**Authors:** Justyna Kasińska, Paweł Malinowski, Piotr Matusiewicz, Włodzimierz Makieła, Leopold Barwicki, Dana Bolibruchova

**Affiliations:** 1 Faculty of Mechatronics and Mechanical Engineering, Department of Metal Science and Materials Technology, Kielce University of Technology, Al. Tysiąclecia Państwa Polskiego 7, 25-314 Kielce, Poland; 2 Faculty of Foundry Engineering, Department of Foundry Processes Engineering, AGH University of Krakow, Al. Mickiewicza 30, 30-059 Krakow, Poland; pamalino@agh.edu.pl; 3 Faculty of Metals Engineering and Industrial Computer Science, Department of Physical and Powder Metallurgy, AGH University of Krakow, Al. Mickiewicza 30, 30-059 Krakow, Poland; matus@agh.edu.pl; 4 Faculty of Mechatronics and Mechanical Engineering, Department of Manufacturing Engineering and Metrology, Kielce University of Technology, Al. Tysiąclecia Państwa Polskiego 7, 25-314 Kielce, Poland; wmakiela@tu.kielce.pl; 5 ENREM-POŁANIEC Sp. z o.o., Tursko Małe 107, 28-230 Połaniec, Poland; leopold.barwicki@gmail.com; 6 Department of Technological Engineering, Faculty of Mechanical Engineering, University of Zilina, Univerzitná 8215/1, 010 26 Zilina, Slovakia; danka.bolibruchova@fstroj.uniza.sk

**Keywords:** matrix replicas, image analysis, stereological measurements, power plants, non-destructive tests

## Abstract

This article introduces a novel approach for assessing microstructure, particularly its degradation after extended operation. The authors focus on creep processes in power plant components, highlighting the importance of diagnostics in this field. This article emphasizes the value of combining traditional microstructure observation techniques with image analysis. A non-destructive method of evaluating microstructure parameters (matrix replicas) is presented, and its accuracy is evaluated against the conventional destructive method. The assessment utilizes quantitative data derived from classical stereological principles and image analysis. Parameters like mean chord length, relative surface area, mean cross-sectional area, and mean equivalent diameter are compared for replica and metallographic specimens. The results show that the replica method accurately reproduces the microstructure. In their conclusions, the authors highlight the importance of developing visual methods alongside the application of artificial intelligence while indicating the challenges in achieving this goal.

## 1. Introduction

The energy industry faces the pressing challenge of meeting rising energy demand while addressing environmental concerns. Power plants, both nuclear and fossil-fuel-based, are at the heart of this challenge. To address this, new energy sources must be explored, and the efficiency of existing plants must be improved [1,2,3,4].

Efficiency improvements can come from various sources, such as automating processes, providing better fuel, and optimizing energy transmission. However, the efficacy of these initiatives is predicated on accurate diagnostics of power plants and energy systems [5].

**Figure 1:**
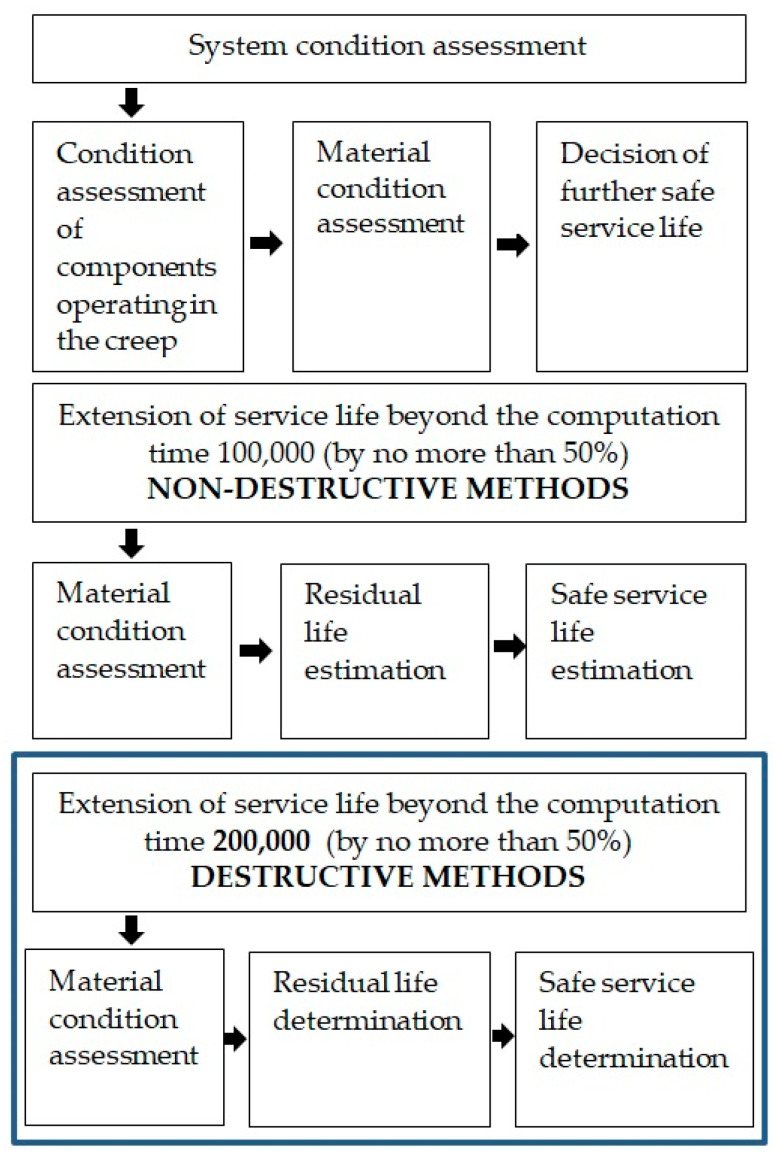
Diagram of diagnostic stages for power-generating unit elements.

**Figure 2:**
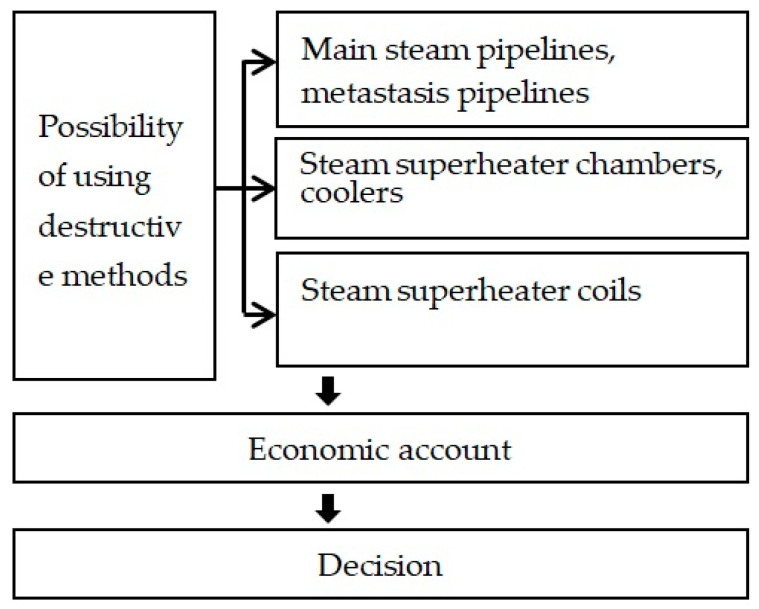
Destructive methods in the decision-making process.

Diagnostics in the energy industry refers to a complete process of evaluating the condition of equipment by analyzing test data and calculations and determining the remaining safe operating life. The assessment of installations in power units is carried out in phases, and the direction of further investigations is contingent upon the information gathered throughout the ongoing process [6,7,8]. Initial assessments identify critical areas, and non-destructive testing provides preliminary insights (although with a significantly smaller scope for inferring the remaining safe operating time). A general overview of the stages is shown in Figure 1. It is important to emphasize that the possibility of using destructive testing is limited to selected components of power plants (Figure 2) and is also very costly. Therefore, economic considerations cannot be ignored throughout the diagnostic process [9]. The key decision about when to conduct destructive testing often depends on initial non-destructive test results. 

Typically, material condition assessment relies on acquiring and interpreting data collected during planned downtime, which includes surface reconstruction, hardness measurements, and deformation analysis. Such data are not readily or routinely incorporated into a predictive model to estimate remaining useful life, and critical decisions regarding operation, repair, or replacement are often based on conservative expert assessments [10,11]. The primary inspection of the parent material is planned to occur at approximately the 50% point of its projected creep rupture life. Bends will be the initial focus of this inspection. The relevant inspection body will evaluate surface replicas using a predefined set of criteria [12], considering factors such as cavitation density and the alignment of grain boundary damage. The accuracy of material assessment based on replica analysis is significantly influenced by the quality of the metallographic specimen preparation. 

The replica technique, which originated in the 1950s [13], has undergone substantial advancements to accommodate a diverse range of materials and observation methods [14,15,16], particularly electron microscopy. Originally employed for non-destructive crack examination [17], the technique was subsequently adapted to extract and analyze particles. 

The hardening of a polymer solution on the surface creates a replica that captures the surface topography. Fisher [13] pioneered the application of this technique to etched metal surfaces for microstructure observation and particle extraction. However, the limitations associated with film thickness for transmission electron microscopy necessitated the development of alternative materials and techniques [18]. Consequently, the replica technique has evolved into two primary categories: extraction replicas for transmission electron microscopy [19,20,21] and surface replicas for both light and scanning electron microscopy. Surface replicas have gained prominence not only for microstructure analysis but also for quantifying surface topography parameters [22,23].

The authors of the present study examined a non-destructive method of assessing the microstructure of steel using the matrix replica method in the context of potentially obtaining more information through the application of modern computer methods, including image analysis. This article includes a detailed analysis of the matrix replica method, along with a quantitative estimation of the accuracy of reproducing the actual microstructure based on the measurements performed. The measurements were based on fundamental parameters used in materials stereology such as mean chord, mean cross-sectional area, and relative surface area.

Furthermore, the authors demonstrated the possibility of combining conventional research methods, such as microstructure observations and measurements of its fundamental parameters, with modern image analysis techniques. This article points to a new path toward material evaluation using elements of artificial intelligence, which, in cases where human-conducted destructive testing is not feasible (e.g., in space), may represent a fundamental solution in the future. 

## 2. In-Service Degradation of Steel Microstructure

Operating conditions in power plants induce microstructural changes in steel components. Creep is a particularly significant phenomenon in this context. It is a destructive process that affects the microstructure of boiler steels and is the primary failure mechanism for materials operating above their homologous temperature. This necessitates investigating microstructural changes occurring in boiler steels during prolonged operation as a result of this process [24,25,26].

Elevated temperatures and pressures significantly alter the original ferritic–perlitic structure [27], leading to perlite degradation through cementite spheroidization while preserving phase proportions [28]. Perlite initially defragments before carbide coagulation and growth occur. Carbide formation at grain boundaries is detrimental. Bainitic steels present a more complex degradation path, with less pronounced microstructural changes, carbide coagulation, and alloying element depletion. Reduced solid solution strength, coupled with creep and microcracking [29], ultimately compromises material integrity and can lead to equipment failure.

Current practices involve comparing microstructures to assess degradation and predict the remaining life of power plant components. Existing steel classifications for energy applications categorize materials based on structural changes without internal damage and those with creep-related damage [30,31]. The expertise and judgment of inspectors, especially in nuclear power plants, significantly influence the application of these methods [32]. This study introduces a novel approach to assess the microstructure of creep-affected steel using quantitative metallography. By non-destructively analyzing surface replicas of components, it is possible to accurately detect the effects of stress concentration (microcracks), which is essential for predicting component life and ensuring operational safety.

## 3. Matrix Replica Method

The matrix replica method is a non-destructive testing technique employed in diagnostic procedures to evaluate the extent of material degradation by comparative analysis. This involves comparing the obtained microstructure image with reference images from existing standards and guidelines, which illustrate the changes in microstructure caused by temperature and stress. These standards provide a collection of images for materials used in power plants, classified by steel grade and microstructure type.

While there are many established protocols for producing surface replicas, mastering this technique, particularly for specific materials, requires a high degree of expertise and experience. The quality of the replica is critically dependent on the quality of the metallographic preparation, making it challenging to develop standardized procedures and limiting its application in predictive modeling for durability assessment. 

**Figure 3:**
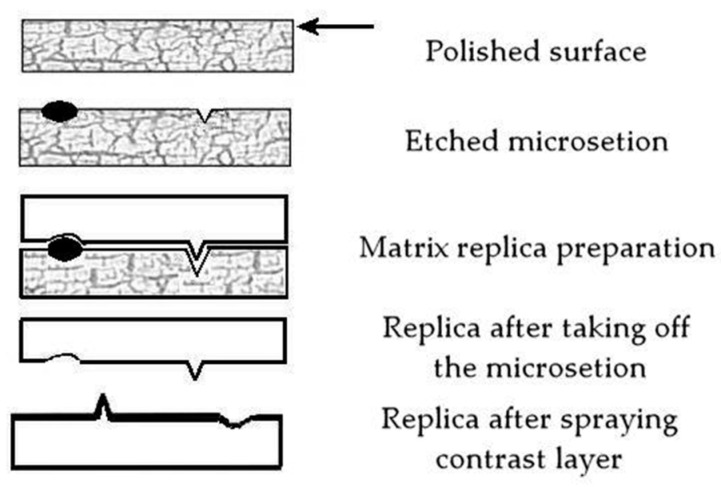
Microstructure mapping using the matrix replica method [9].

**Figure 4:**
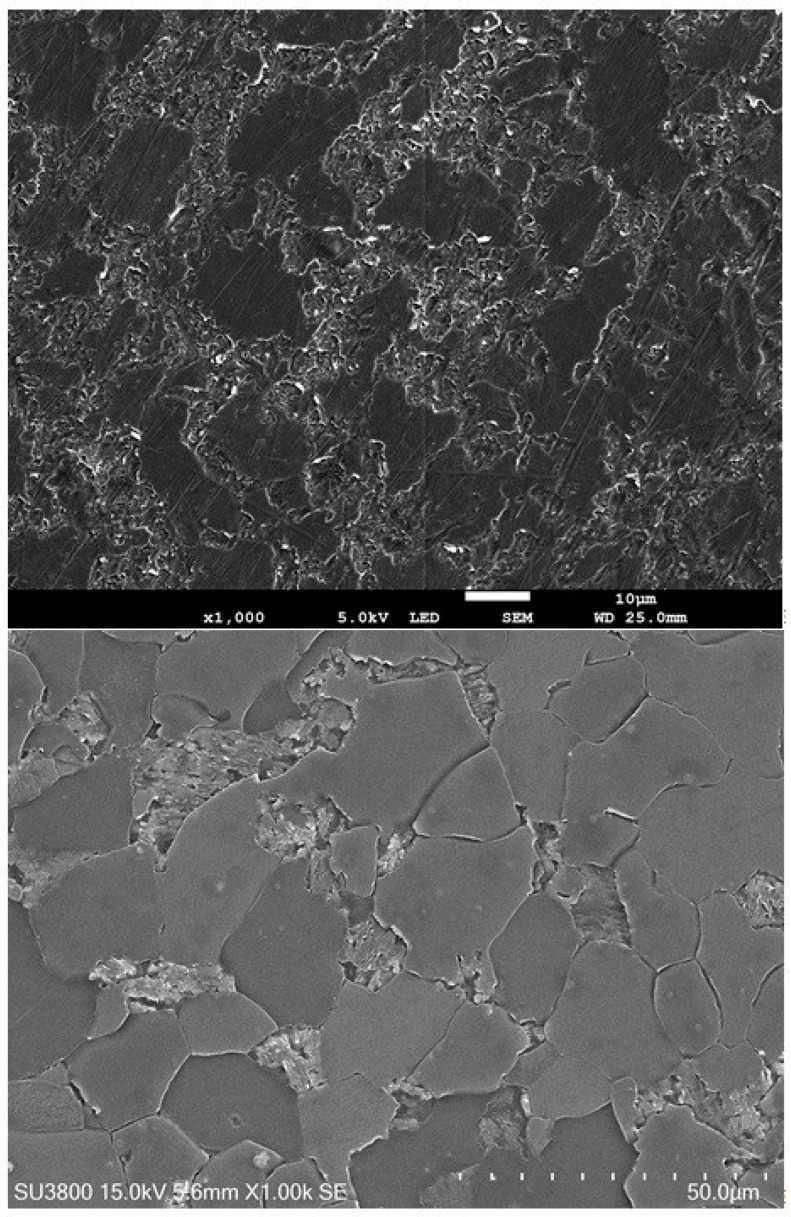
Example of replicas: the upper one, made on an incorrectly prepared surface with visible grinding marks, and the lower one, correctly prepared on 15HM steel, ferrite, and perlite grains with a retained lamellar cementite structure.

Microstructure analysis of ferritic–perlitic, ferritic–bainitic, and martensitic steels relies on light and scanning electron microscopy, utilizing both metallographic specimens and matrix replicas from industrial components. Matrix replicas, created by applying a thin polymer film (triafol) to the prepared and etched surface, capture a detailed impression of its topography, offering a non-destructive “mirror image” of the microstructure (Figure 3) [9]. The replica method, widely employed in the power industry, is crucial for predicting component life [33]. However, replica quality, inspector expertise, and the limitations of descriptive microstructural analysis introduce uncertainties in assessing degradation rates (Figure 4). 

**Figure 5:**
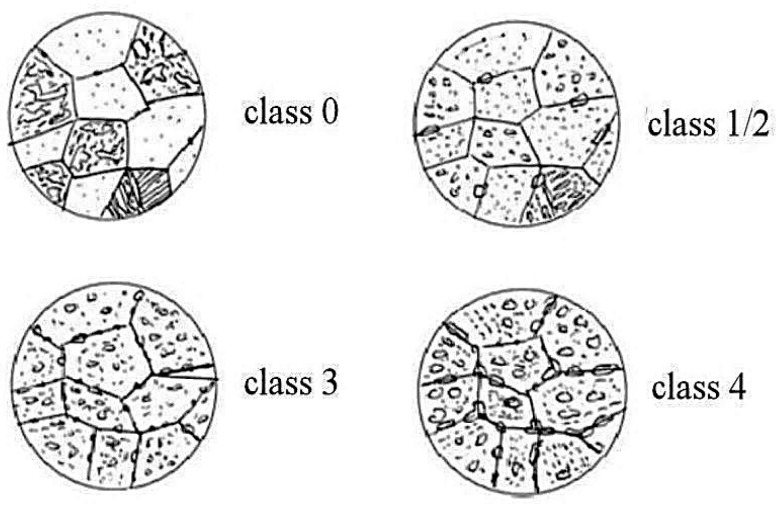
Microstructure patterns and assigned classes acc. to UDT Guidelines [34].

In Poland, the Guidelines of the Office of Technical Inspection [34] are the basis for assessing power-generating unit components. The first stage of the assessment consists of assigning the main class to the observed microstructure. Figure 5 shows the classes of microstructures according to the guidelines. The corresponding descriptions are given in Table 1.

Similar standards/instructions are used in other countries:

NT NDT 010 [35]: High Temperature Components In Power Plants: Remnant Lifetime Assessment, Replika Inspection published by NORDTEST Finland 1991-05.

ASTM E1351-01 [36]: Emergency Standard Practice for Production and Evaluation of Field Metallographic Replica.

Alstom instruction: Materials Technology Center Chattanooga, TN 423-752-2946.

Instructions for using the polymer film: SPI Replicating Tapes and Sheets, West Chester, PA 19380.

Various quantitative models have been explored to assess microstructure based on replica data. To minimize the influence of metallographic preparation, researchers have introduced parameters designed to reduce the method’s sensitivity to surface conditions. Shammas [37,38], for example, proposed parameter “A”, which quantifies the fraction of grain boundaries affected by cavitation or cracking. Despite the requirement for accurate grain boundary and damage detection, this approach has laid the groundwork for quantitative microstructure evaluation. Although these early models exhibited a conservative bias and have undergone subsequent modifications [39], they highlight the potential of quantitative approaches. However, further refinements are necessary for practical industrial implementation. Nonetheless, the concept of quantifying metal damage through various measurements remains an intriguing avenue of research [40].

## 4. Image Analysis

Image analysis refers to the process of extracting information from an image, where the input is an image, but the output can take various forms, such as numerical values, arrays, text, decisions, or actions. Image processing, on the other hand, involves transforming an image into another image. Consequently, image analysis and processing are interconnected, and image processing is often a prerequisite for extracting meaningful features from an image. 

To enhance the objectivity of microstructure degradation assessment, novel solutions can be explored using artificial intelligence tools. While metallographic specimens and matrix replicas remain essential, high-quality microscopic images (LM, SEM) are paramount. The images, captured in binary, grayscale, or color formats, undergo a structured analysis process:

- Pre-processing;
- Segmentation;
- Feature recognition and analysis;
- Classification and interpretation.

Pre-processing includes the following:

- Geometric transformation for correcting optical errors;
- Context-free transformation for modifying successive image points using logical and arithmetic operations, usually without considering the condition of the neighboring points. Typical point operations include binarization (which involves converting a color or grayscale image into a black-and-white image consisting of only two levels of intensity (binary)), negativizing, brightening, or dimming of the image;
- Context-based transformations for modifying the sequential elements of an image depending on their condition and their surroundings;
- Spectral transformations employing the Fourier transform (transformation in the image spatial frequency domain) to alter the input data structure;
- Morphological transformations for modifying only a selected part of the image, the environment of which is consistent with the structural element. Image analysis offers an effective method for accurately and quantitatively describing microstructures and their defects.

Image segmentation is a technique that divides an image into distinct regions based on specific characteristics. These regions, composed of groups of pixels, are homogeneous in terms of properties like grayscale level, color, or texture. Segmentation is essential for identifying object boundaries (grains or phases in a microstructure). The pre-processing step involves identifying and retaining the most relevant features while discarding redundant or irrelevant ones. By focusing on the most informative features, one can effectively address excessive dataset challenges. Future selection uses information theory, precisely mutual information, to quantify the relationship between different features, which allows for prioritizing the most relevant features for subsequent analysis [41,42].

Classification is a stage in image recognition where collected information about an object is used to assign it to a predefined group. The system must make an accurate interpretation based on extracted features and categorize the object into the appropriate class. For example, in microstructures, these classes might include inclusions, voids, cracks, grain boundaries, etc.

Table 2 illustrates clearly that this method significantly outperforms traditional human visual assessment [43].

## 5. Materials and Methods

### 5.1. Experimental Section

**Figure 6:**
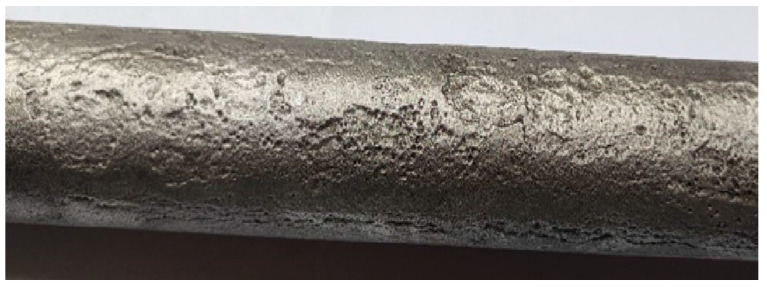
A fragment of a steam pipeline used to create the replicas.

A 13HMF steel specimen from a primary steam pipeline was selected to measure microstructure parameters (Figure 6). The chemical composition of 13HMF steel is as follows: 0.18% C; 0.3% Si; 0.7% Mn; 0.5% Cr; 0.53% Mo; 0.15% Ni; 0.011% Al; 0.015% S; 0.022% P). The pipeline time in service was 230,000 h at an operating pressure of 13.8 MPa and an operating temperature of 540 °C. A metallographic specimen (MS) was prepared by grinding, using variable gradation abrasive papers and polishing, and etched in a solution of HNO_3_ in C_2_H_5_OH. Then, ten areas of 400 × 400 μm were marked on the prepared MS. A matrix replica (MR) was prepared and coated with a gold layer. Observations of both variants were carried out in a JSM-7100F field-emission scanning electron microscope (JEOL Ltd., Tokyo, Japan), searching for identical areas (Figure 6). This procedure aimed to minimize errors due to differences between the microstructures. MS observations were carried out at an accelerating voltage of 15 kV, while on the MR the voltage was 5 kV. The reduced value for the replica is due to polymer film deformation at higher accelerating voltages caused by electron interactions. The deformation does not occur in the steel sample (MS). The adjusted voltages facilitate accurate microstructure observation.

### 5.2. Measuring Methods

**Figure 7:**
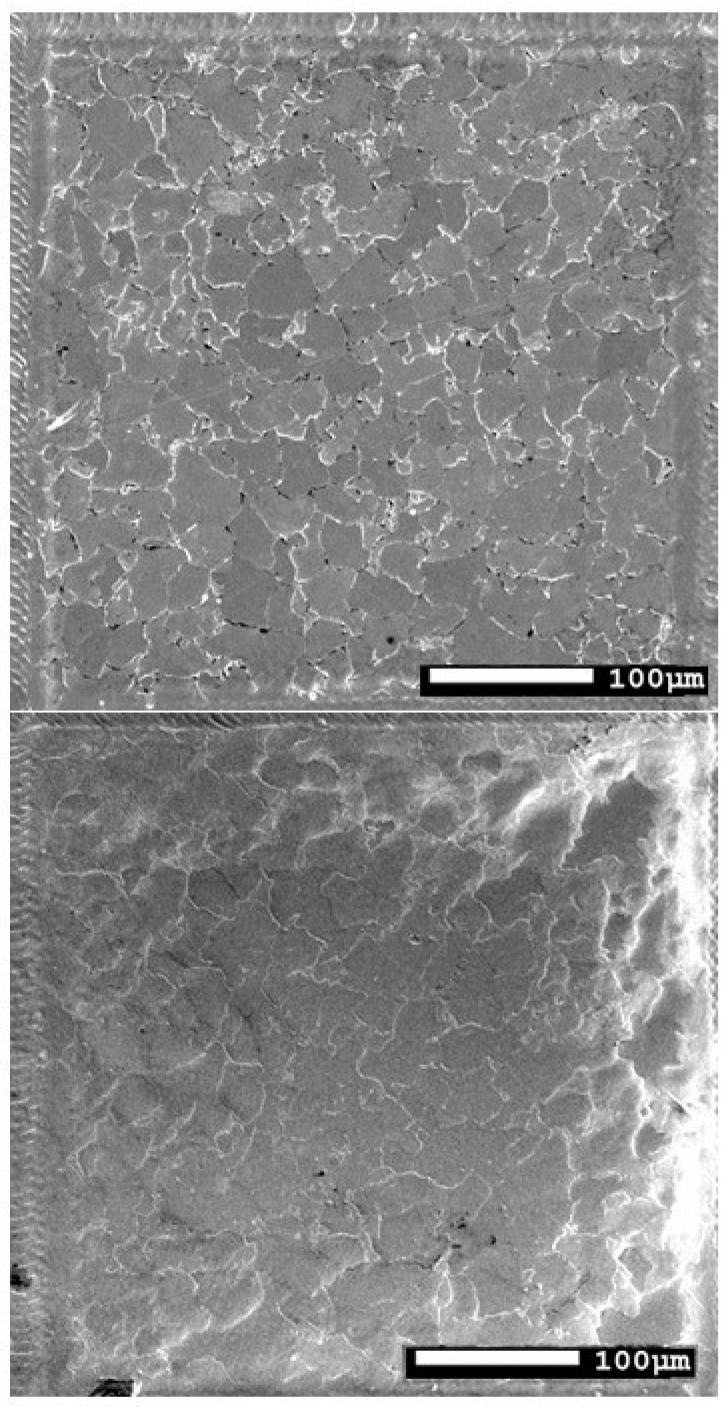
Microstructure of 13HMF steel on the metallographic sample (**top**) and on the replica (**bottom**).

Grain size measurement, a prevalent quantitative metallographic technique, was selected to compare observations from metallographic specimens (MSs) and matrix replicas (MRs) (Figure 7). It is important to acknowledge a limitation: grain size measurements are performed on two-dimensional images, while metal grains are three-dimensional structures measured on planar images. This can introduce some discrepancies. Grain size can be quantified using various parameters, including the mean grain cross-sectional area, the mean number of grains per unit area, the mean diameter of a grain, or the mean grain chord length. Additionally, comparison procedures exist that utilize pattern charts. These charts classify microstructures based on visual similarity, assigning a group number to the observed microstructure.

#### 5.2.1. Intercept Method

The method of randomly running test lines allows the determination of the relative grain boundary surface area S_V_. In the case of single-phase structures, Equation (1) is used:

S_V_ = 2P_L_ ≈ 2N_L_ [mm^−1^] (1)

where

P_L_—mean number of grain boundary intersections by the randomly placed test lines with grain boundaries per unit length of test lines;

N_L_—mean number of grains (relative number of chords) per unit length of test lines.

The above formula is applied to isometric systems of boundary surfaces. The mean linear chord length Ī is taken as a grain size measure.

Ī = L_L_/N_L_ [mm] (2)

where

L_L_ is the relative chord length.

The mean chord length of a flat grain can then be determined by measuring the chord lengths of the specified number of grains. The second technique is to count the grains n intersected by the test line of a length L_T_, given by parameters P_L_ or N_L_:

Ī = L_T_/n = 1/N_L_ = 1/P_L_ (3)

Formulas (1) and (3) reveal a relationship between the relative surface area S_V_ of intergranular boundaries in a single-phase structure and the mean chord length (derived from relation P_L_ = ½S_V_). This relationship is expressed in the following formula:

S_V_ = 2/Ī

**Figure 8:**
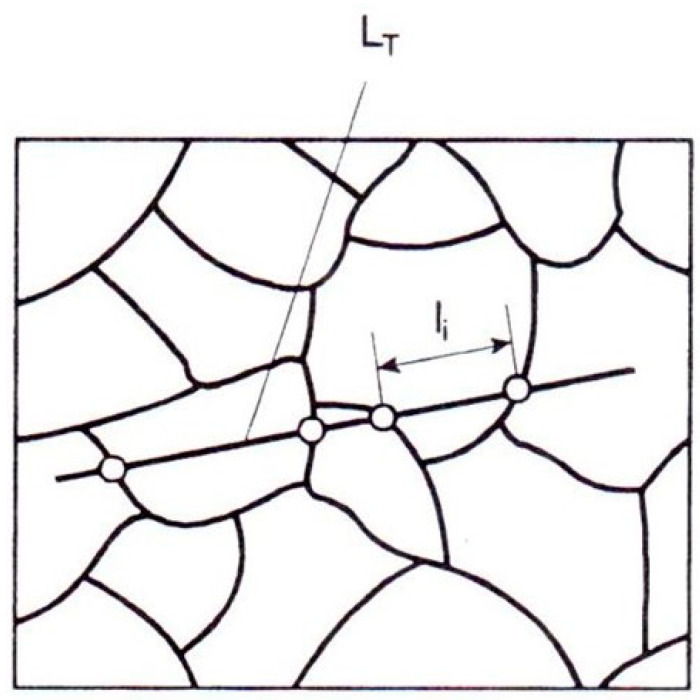
Chords in a single-phase alloy [44].

Grain size determination using the mean chord method involves counting the grains intersected by test lines on a photomicrograph. Edge grains at the line ends are counted as one grain (Figure 8) [44].

#### 5.2.2. Planimetric Method

**Figure 9:**
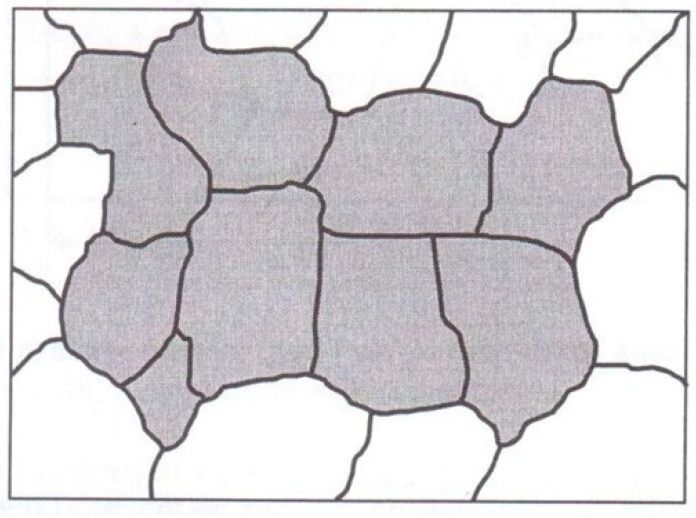
Mean grain area measurement using the planimetric method [44].

The planimetric method is considered the most precise approach for grain size determination. It involves measuring the surface area of individual grains within a designated test area using a planimeter. The mean number of grains per unit area is then calculated by dividing the total number of grains by the actual measured area (expressed in mm^2^ or μm^2^) (Figure 9) [44].

The choice of method to determine microstructure parameters will depend on its type and the quality of the acquired images. If it is impossible to unambiguously determine the grain or interphase boundaries, the linear intercept method should be used, which will help avoid making a significant measurement error. In the case of proper sample preparation and correct imaging, the planimetric method will allow for obtaining more information, such as shape factors or relative surface area of grain cross-sections. 

### 5.3. Image Analysis Measurements

**Figure 10:**
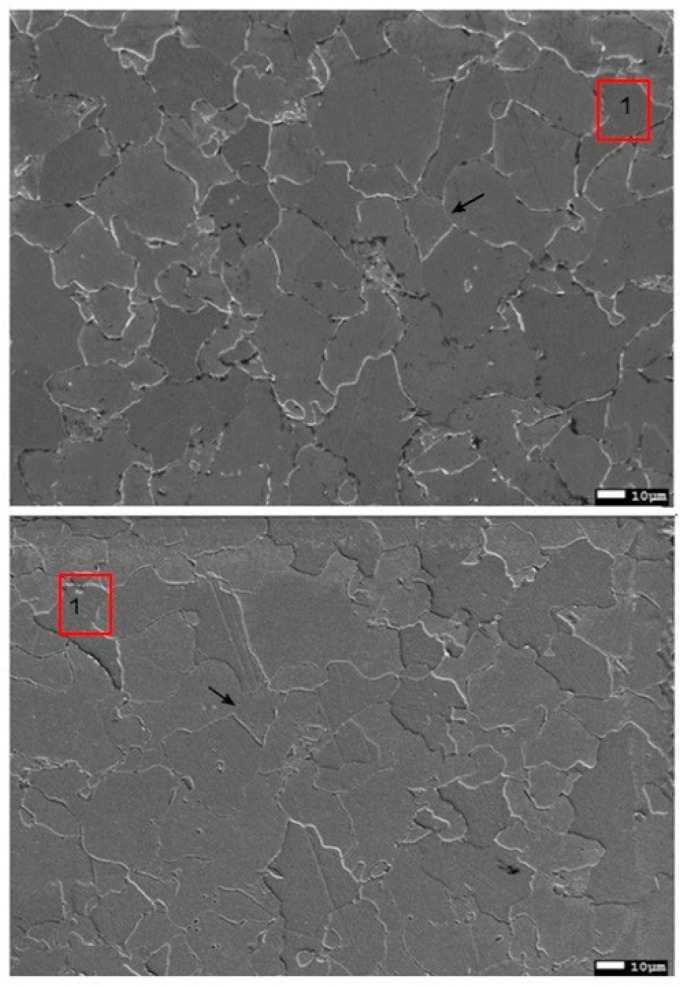
SEM images of the specimen (**top**) and replica (**bottom**) with marked characteristic features.

**Figure 11:**
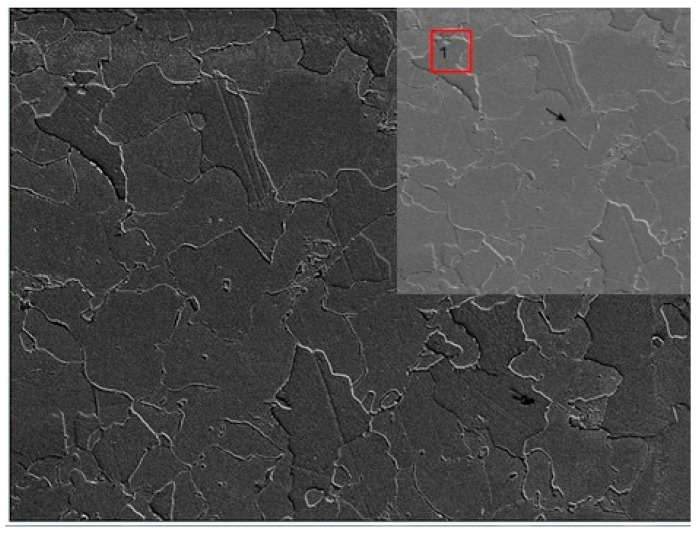
Replica image after checking Adjust → Brightness/Contrast.

**Figure 12:**
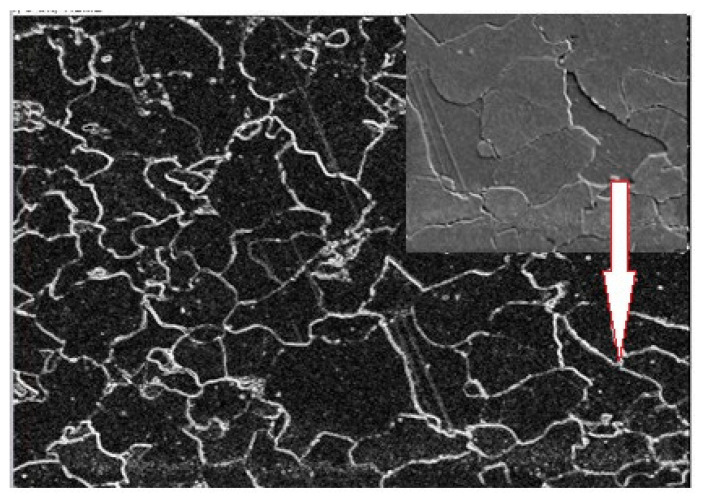
Use of the “Find Edges” feature.

The initial step in image analysis involved locating identical areas on both the metallographic specimen (MS) and matrix replica (MR) at the same magnification (Figure 10). This process relied on identifying a characteristic grain, such as point “1” in Figure 10. However, accurately replicating the microstructure from the MS to the MR image, particularly grain boundaries, presented a challenge due to poorly defined boundaries in the MR image. To address this problem, the ImageJ software (https://imagej.net) “Adjust” function was utilized, followed by “Brightness/Contrast” (Figure 11). In areas where grain boundaries remained unclear, the “grain detection” option was applied (Figure 12). 

Grain size was determined by measuring the mean grain chord length on the prepared images. First, the images were calibrated by measuring the actual length of the scale marker and its corresponding pixel count. 

**Figure 13:**
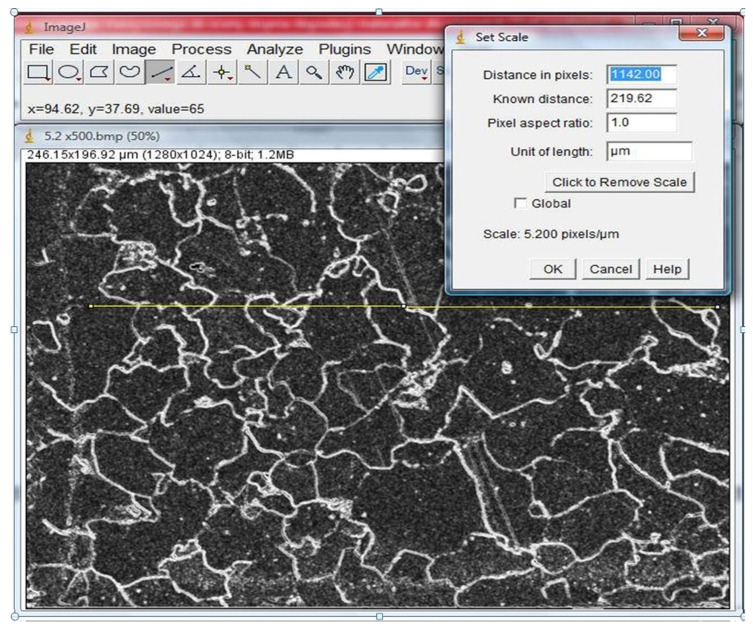
Placing test lines and reading their actual length.

Next, following the intercept method, test lines were overlaid onto the MS and MR images (Figure 13). The number of grains intersected by each line was then counted. The mean chord length on the i-th test line ${\bar{l}}_{i}$ was calculated using Equation (3). This process was repeated for 100 measurements on both the metallographic specimen (MS) and corresponding matrix replica (MR) areas. All measurements were recorded in Excel.

The grain area in the microstructure images for MS and MR was measured according to the description provided in the Section 5.2.2. 

## 6. Results and Discussion

**Figure 14:**
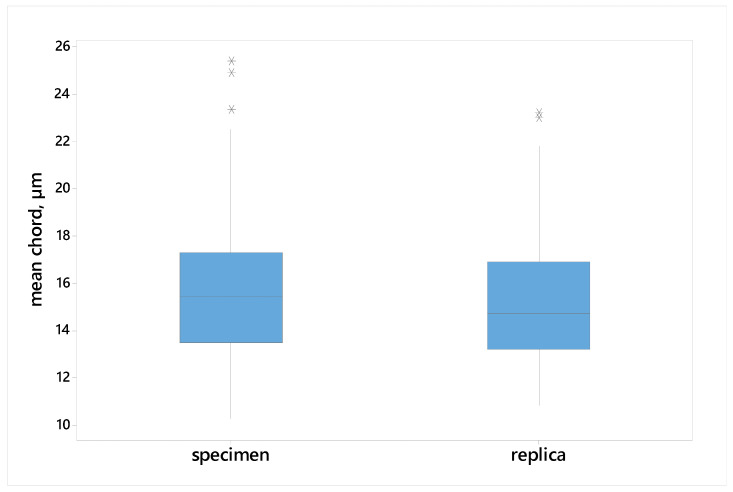
Box plots of the mean chords measured on the specimens and replicas. *—position of extreme values.

**Figure 15:**
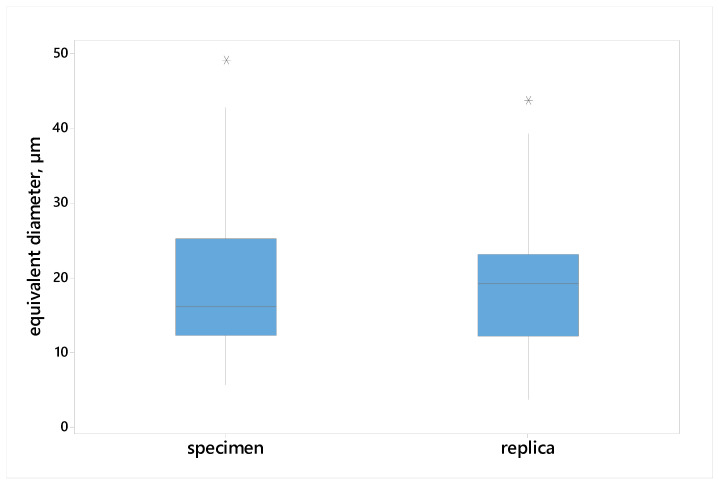
Box plots of equivalent diameters measured on the specimens and replicas. *—position of extreme values.

**Figure 16:**
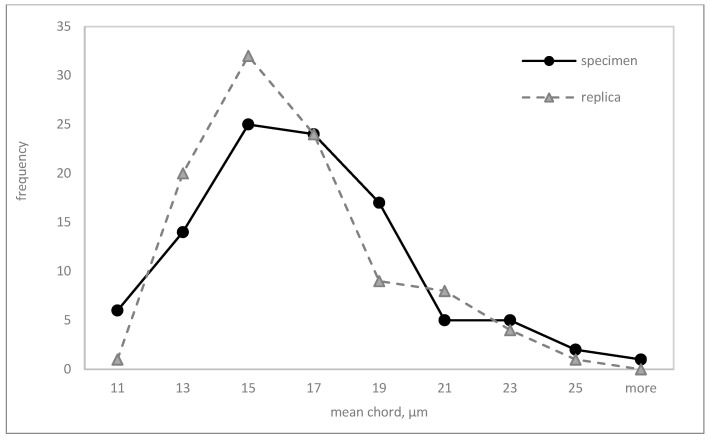
Mean chord distributions measured on the specimens and replicas.

**Figure 17:**
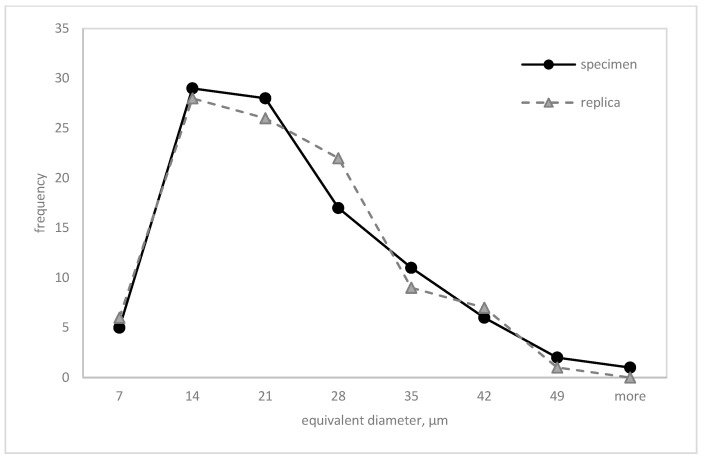
Equivalent diameter distributions measured on the specimens and replicas.

An extensive statistical analysis was performed to evaluate the equivalence of microstructure parameter measurements obtained from metallographic sections (MSs) and matrix replicas (MRs). This analysis included descriptive statistics (Table 3), graphical representations (box plots (Figure 14 and Figure 15) and frequency polygons (Figure 16 and Figure 17)), and hypothesis testing. Parametric tests (*t*-test for means, F-test for variances) and a non-parametric test (Mann–Whitney U test) were employed to assess the equality of means, variances, and distributions, respectively. 

Inference regarding the stated hypotheses was conducted at a significance level of α = 0.05. This means there is a 5% probability of rejecting a true null hypothesis. The significance level of α = 0.05 is a standard value for statistical errors. Conversely, α = 0.05 implies that the statistical inference is performed at a confidence level of 1 − α = 0.95, providing 95% confidence in the analysis results [45].

The outcome of a statistical hypothesis test is a *p*-value. If the calculated *p*-value is less than or equal to the predetermined significance level, α, the null hypothesis is rejected. Conversely, if the *p*-value is greater than α, there is insufficient evidence to reject the null hypothesis. From a practical standpoint, failing to reject the null hypothesis is often interpreted as accepting it [45].

Statistical analysis was performed on individual grain size measurements (mean chord length and cross-sectional area) to determine average values and standard deviations. These values were then used to calculate additional grain size measures, such as the relative surface area (S_V_) and equivalent diameter ($\bar{d}$) (diameter of a circle with an area equal to the grain), which are presented in Table 3. The results of the statistical tests (*p*-values) are presented in Table 4. 

Figure 14 and Figure 15 compare mean grain size (mean chord length and equivalent diameter) obtained from MS and MR images using box plots. No significant differences in either the center position or the spread of the data between the two measurement methods were observed. These observations were supported by the results of the statistical tests (*t*-test for means and F-test for variances). The *p*-values exceed the significance level of 0.05, indicating that there is insufficient evidence to reject the null hypotheses (Table 4). 

Figure 16 and Figure 17 show the empirical distributions of the mean chord lengths and equivalent diameters. Results of the Mann–Whitney U test for homogeneity supported the prior findings. No statistically significant differences between the distributions of measurements from MS and MR techniques were found at a significance level of α = 0.05 (Table 4).

Grain size is one of the key microstructure parameters that can be used to assess the degradation rate of power plant components after long-term operation. It was found that grain size assessment can effectively be carried out by measurements on matrix replicas (MRs) and metallographic specimens (MSs). When employing intercept counting and planimetric methods, no statistically significant differences were found in the mean chord, relative area, or equivalent diameter between the two methods. The minor discrepancies observed might be attributed to replica shrinkage.

## 7. Conclusions

This analysis of the 13HMF steel microstructure following long-term service underscores the value and applicability of computer image analysis for quantifying microstructural features. This study demonstrated that grain size determination could be effectively achieved using measurements from both metallographic specimens (MSs) and matrix replicas (MRs). Comparisons of mean chord length and equivalent diameter revealed statistically insignificant differences between the MS and MR measurements, suggesting that matrix replica analysis can be a reliable non-destructive technique for grain size assessment. 

The research and analyses conducted in this study led to the following key findings:

- The replica method accurately reproduces the real microstructure with 95% precision, which can serve as a basis for the correct determination of quantitative parameters using this non-destructive method;
- Image analysis combined with a semi-automated measurement methodology is a suitable tool for determining the quantitative characteristics of microstructure images;
- The matrix replica method, as a non-destructive method, will allow for a reduction in the costs of studies where samples must be taken for microstructure observation.

The replica method offers a non-destructive approach to detecting creep damage at grain boundaries in thick-walled components. By eliminating the need for sampling, this method provides a cost-effective means of assessing the condition of components such as chambers and pipelines. For components that have exceeded 200,000 h of service, this method is mandatory. Quantitative analysis using the replica method can inform decisions about the need for further, more destructive testing, such as accelerated creep tests, thereby optimizing diagnostic costs. 

By incorporating quantitative parameters into the assessment of microstructure degradation in power plant components, subjectivity in evaluations can be minimized. Substituting destructive metallographic sampling for non-destructive matrix replica analysis, including grain size measurements, is a crucial step toward optimizing diagnostic processes. By automating and robotizing industrial processes, the need for human intervention in hazardous environments, such as confined spaces like pressure vessels and pipelines, can be eliminated. This shift toward automation necessitates a multidisciplinary approach to diagnostics, encompassing materials science and other relevant fields, which the authors wish to highlight. The material analysis techniques presented in this paper can be integrated into metallurgical processes to improve quality control and enable predictive modeling of material properties. The material analyses proposed in this article can be implemented in metallurgical processes in the future (for castings, welds, or plastically deformed materials). Real-time process monitoring typically relies on process parameter control, but the introduction of microstructure analysis at the quality-control stage will allow for the creation of comprehensive databases on material quality and enable the prediction of material properties.

Looking to the future, image analysis as a research tool has been developing for several decades. Advanced computers have significantly increased the resolution of digital images and processing power compared to past technologies. Diverse image formats like JPG, GIF, and BMP can be analyzed across various operating systems such as Windows and Linux [46].

Building on this foundation, the future of microstructure evaluation holds great promise. Direct observation using visual techniques (cameras, scanners) has the potential to replace the replica method, offering even greater accuracy by eliminating film shrinkage. However, the integration of artificial intelligence into microstructure evaluation presents significant challenges. Training AI models requires extensive datasets, which can be time-consuming and resource-intensive to acquire. Nevertheless, the eventual goal of AI-aided microstructure analysis may be achievable by breaking down the task into smaller, manageable steps.

## Figures and Tables

**Table 1:** Microstructure classes based on degradation types.

Class	Degradation Type
0	A mixture of ferrite and bainite with a proportion of perlite
1, 2	Coagulation of precipitates in bainite, numerous fine precipitates evenly distributed in ferrite, and a few considerably sized precipitates at the boundaries of ferrite grains
3	Almost complete disappearance of bainitic areas and chains of large precipitates at the boundaries of ferrite grains
4	Coagulated carbides in the ferrite and chains of large precipitates at the boundaries of ferrite grains

**Table 2:** Benefits and drawbacks of using image analysis.

Feature Analyzed	Traditional Analysis: Human Visual System	Computer-Aided Image Analysis
Fatigue after extended work	Highly sensitive	Insensitive
Sensitivity to illusion	Highly sensitive	Insensitive
Required image quality	Mean quality may be acceptable for quantitative analysis	Images for automatic image analysis must comply with standards of the highest quality
Repeatability of results	Low	Full repeatability of automatic analysis and high repeatability in semi-automatic analysis
Reproducibility of results	Low	Full
Qualitative evaluation of the microstructure	Can be very good	Poor and difficult to automate
Quantitative analysis of the microstructure	Time-consuming, some parameters cannot be determined	Can be very good
Analysis costs	Relatively low when analyzing single specimens, increases rapidly as the number of specimens increases	High for single-specimen analysis, drops significantly for bulk, routine analysis

**Table 3:** Grain sizing results.

	Mean Chord	Standard Deviation of Chords	Relative Surface Area	Mean Cross-Sectional Area	Standard Deviation of the Cross-Sectional Area	Mean Equivalent Diameter
	$\bar{l}$, µm	$s_{l}$, µm	$S_{V}$, mm^2^/mm^3^	$\bar{a}$, µm^2^	$s_{a}$, µm	$\bar{d}$, µm
Specimen (MS)	15.78	3.22	126.7	373.4	378.0	19.38
Replica (MR)	15.39	2.84	130.0	352.4	321.5	19.16

**Table 4:** Statistical test results.

	Mean Chord	Mean Equivalent Diameter
Test	*p*-Value	
*t*-test for means	0.364	0.874
F-test for variances	0.108	0.157
Mann–Whitney U test for distributions	0.319	0.902

## Data Availability

The original contributions presented in this study are included in the article. Further inquiries can be directed to the corresponding author.
